# The impact of the lookback period and definition of confirmatory events on the identification of incident cancer cases in administrative data

**DOI:** 10.1186/s12874-017-0407-4

**Published:** 2017-08-14

**Authors:** Jonas Czwikla, Kathrin Jobski, Tania Schink

**Affiliations:** 1 Department of Health, Long-term Care and Pensions, SOCIUM Research Center on Inequality and Social Policy, University of Bremen, P.O. Box 33 04 40, 28334 Bremen, Germany; 2 High-Profile Area Health Sciences, University of Bremen, P.O. Box 33 04 40, 28334 Bremen, Germany; 3 Department of Health Services Research, Carl von Ossietzky University of Oldenburg, P.O. Box 2503, 26111 Oldenburg, Germany; 4 Leibniz Institute for Prevention Research and Epidemiology - BIPS, Drug Safety Unit, Achterstrasse 30, 28359 Bremen, Germany

**Keywords:** Neoplasms, Breast neoplasms, Prostatic neoplasms, Colorectal neoplasms, Incidence, Administrative claims, Validation, International classification of diseases, Epidemiology, Health services research

## Abstract

**Background:**

This cohort study examined the impact of the lengths of lookback and confirmation periods as well as the definition of confirmatory events on the number of incident cancer cases identified and age-standardized cumulative incidences (ACI) estimated in administrative data using German cancer registry data as a benchmark.

**Methods:**

ACI per 100,000 insured persons for breast, prostate and colorectal cancer were estimated using BARMER Statutory Health Insurance claims data. Incident cancer cases were defined as having an in- or outpatient diagnosis in 2013, no diagnosis in a lookback period of 1 year and a second diagnosis (or death) in a confirmation period of 1 quarter. We varied lookback periods from 1 to 7 years, confirmation periods from 1 to 4 quarters as well as the definition of confirmatory events and compared ACI estimates to cancer registry data.

**Results:**

ACI were higher for breast (138.7) and prostate (103.6) but lower for colorectal cancer (42.1) when compared to cancer registries (119.3, 98.0 and 45.5, respectively). Extending the lookback period to 7 years reduced ACI to 129.0, 95.1 and 38.3. An extended confirmation period of 4 quarters increased ACI to 151.3, 114.9 and 46.8. Including breast and colorectal surgeries as a confirmatory event reduced ACI to 114.9 and 37.1, respectively.

**Conclusions:**

The choice of lookback and confirmation periods and the definition of confirmatory events have considerable impact on the number of incident cancer cases identified and ACI estimated. Researchers need to be aware of potential misclassification when identifying incident cancer cases in administrative data. Further validation studies as well as studies using administrative data to estimate cancer incidences should consider several choices of the lookback and confirmation periods and the definition of confirmatory events to show how these parameters impact the validity and robustness of their results.

**Electronic supplementary material:**

The online version of this article (doi:10.1186/s12874-017-0407-4) contains supplementary material, which is available to authorized users.

## Background

Cancers are one of the leading causes of morbidity and mortality worldwide [1] and, in epidemiological studies, often the outcome of interest or an important confounder. To obtain valid results in these studies, it is important to distinguish incident from prevalent and recurrent cancer cases. In recent years, administrative claims data have become an increasingly important source of large, longitudinal data that can be effectively used for epidemiological research. Even though some of the databases allow for long follow-up periods, administrative claims data are left-censored and usually provide no information whether a cancer diagnosis is incident, prevalent or recurrent. However, algorithms based on in- and outpatient claims data for diagnoses and health care provision were developed to identify incident cancer cases [2–9]. The sensitivity, specificity and positive predictive values (PPV) of these algorithms varied considerably and depended not only on the specific cancer site, but also on the complexity of the algorithm used [10]. Moreover, a systematic review including 84 studies published between 1980 and 2013 showed that most researchers built their own algorithms to identify incident breast, prostate and colorectal cancer cases in primary care databases without, however, giving detailed explanations regarding their methods used [11].

To build a valid claims-based algorithm, two issues have to be considered. It is important to (1) identify all cases of the cancer site achieving a high sensitivity and specificity and (2) to distinguish incident from prevalent and recurrent cases. In our previous study, we analyzed the coding quality for outpatient breast, prostate and colorectal cancer diagnoses in German Statutory Health Insurance (SHI) claims data. We demonstrated that a proportion of outpatient cancer diagnoses ranging from 16 to 28% for breast, from 16 to 25% for prostate and from 24 to 32% for colorectal cancer remains unconfirmed depending on the internal validation algorithm applied [12]. This may be due to physicians who also code suspected diagnoses, especially if patients are referred to a specialist. Therefore, in most algorithms, outpatient diagnoses need to be internally validated by a second diagnosis, indicators for therapy or death within a predefined confirmation period [4, 13]. If stricter confirmation criteria are chosen, the specificity and PPV increase, whereas sensitivity might decrease considerably [6, 14]. To distinguish incident from prevalent and recurrent cancer cases, usually, a predefined lookback period without a respective cancer diagnosis is required [15]. However, the longer the lookback period the greater the number of cases that cannot meet the requirement as not enough observation time before the case is available in the database. On the other hand, the shorter the lookback period the greater the number of prevalent and recurrent cases falsely being included as incident.

As, to our knowledge, this has yet not been systematically studied, the aim of this study was to examine the impact of (1) the length of the lookback period, (2) the length of the confirmation period and (3) the definition of confirmatory events on both the number of incident cancer cases identified and cumulative incidences estimated in administrative claims data using German cancer registry data as a benchmark at the population level.

## Methods

### Data source

In Germany, approximately 70 million people (90% of the total population) are covered by the SHI and insured with one of currently 113 (April 2017) SHI funds. The BARMER, which insures more than 9 million people from all regions in Germany, is one of the two largest German SHI funds. Its claims data comprises demographic information for each insured person as well as information on in- and outpatient care. All diagnoses are coded according to the German Modification of the International Classification of Diseases, 10th Revision (ICD-10-GM). The exact date of diagnosis is available for inpatient diagnoses. Outpatient diagnoses can only be assigned quarterly. Since 2004, in Germany, additional coding of diagnostic certainty, which differentiates between G (certain), V (suspected), Z (status post, i.e. (asymptomatic) status after a previous diagnosis) and A (diagnosis excluded) is mandatory for outpatient diagnoses. For data on health care provision which is encoded according to the German uniform assessment standard (EBM) and the German Procedure Classification (OPS), exact dates are available.

### Study design and population

We performed a retrospective cohort study based on claims data of the BARMER covering the years from 2006 to 2014. The study population comprised all insured persons with at least 7 years (or at least 1 year in a sensitivity analysis) of continuous insurance (i.e. no insurance gaps of more than 28 days) on 01 January 2013. Insured persons with missing or invalid information on sex, year of birth or place of residence were excluded. Insured persons who resided outside of Germany were also excluded as they (1) are not captured by the German cancer registries and thus not included in our benchmark data and (2) might receive care outside the German SHI.

### Case definition

We chose the three most common incident cancer sites in Germany, i.e. breast (most common in women), prostate (most common in men) and colorectal (2nd most common in women and 3rd most common in men) cancer, and used ICD-10-GM codes applied by the German Centre for Cancer Registry Data (ZfKD), namely: C50 for breast (women only), C61 for prostate (men only) and C18-C21 for colorectal cancer (women and men) [16, 17]. Incident cancer cases were identified on a quarterly basis considering outpatient diagnoses coded as “certain” and hospital discharge diagnosis reflecting the reason for hospitalization. In the lookback period for identifying prevalent and recurrent cases as well as in the confirmation period for identifying confirmatory events, outpatient diagnoses coded as “status post” and ancillary hospital diagnoses were also considered.

For the baseline algorithm, incident cases were defined as all insured persons with:

- A breast, prostate or colorectal in- or outpatient diagnosis in 2013,
- no respective in- or outpatient diagnosis within the lookback period of 1 year (4 quarters) preceding the index quarter and
- a confirmatory event defined as a second respective in- or outpatient diagnosis (or death) within a confirmation period of 1 quarter following the index quarter.

Insured persons with two incident cancers (breast and colorectal for women; prostate and colorectal for men) in 2013 were counted in each entity.

### Algorithms

To assess the impact of the length of the lookback period, the length of the confirmation period and the definition of confirmatory events on both the number of incident cancer cases identified and cumulative incidences estimated, we varied the baseline algorithm as follows:

- Lookback period: 1 to 7 years.
- Confirmation period: 1 or 4 quarters.
- Definition of confirmatory events:
  ○ exclusion of death as a confirmatory event,
  ○ inclusion of surgery as a required confirmatory event (lumpectomy and mastectomy for breast cancer and endoscopy and colorectal surgeries for colorectal cancer),
  ○ no confirmatory event required.

This resulted in 15 algorithms, which are shown in Table 1.

**Table 1:** Numerator (n), crude cumulative incidences (CCI) and age standardized cumulative incidences (ACI) per 100,000 insured persons for breast, prostate and colorectal cancer in 2013

Algorithm	Description	Breast cancer (women) Denominator: *n* = 4,093,251			Prostate cancer (men) Denominator: *n* = 2,670,298			Colorectal cancer (overall) Denominator: *n* = 6,763,549		
		Num. (n)	CCI per 100,000	ACI per 100,000	Num. (n)	CCI per 100,000	ACI per 100,000	Num. (n)	CCI per 100,000	ACI per 100,000
1. L1-C1	Baseline algorithm, lookback 1 year, confirmation 1 quarter	10,312	251.9	138.7	6200	232.2	103.6	6513	96.3	42.1
2. L2-C1	Lookback 2 years	9826	240.1	133.5	5916	221.5	99.4	6172	91.3	40.1
3. L3-C1	Lookback 3 years	9646	235.7	131.6	5790	216.8	97.6	6011	88.9	39.2
4. L4-C1	Lookback 4 years	9548	233.3	130.6	5730	214.6	96.6	5940	87.8	38.8
5. L5-C1	Lookback 5 years	9478	231.6	129.8	5690	213.1	96.1	5887	87.0	38.5
6. L6-C1	Lookback 6 years	9439	230.6	129.3	5646	211.4	95.4	5857	86.6	38.3
7. L7-C1	Lookback 7 years	9409	229.9	129.0	5623	210.6	95.1	5842	86.4	38.3
8. L1-C4	Confirmation 4 quarters	11,199	273.6	151.3	6884	257.8	114.9	7213	106.6	46.8
9. L7-C4	Lookback 7 years, confirmation 4 quarters	9866	241.0	136.0	6077	227.6	102.9	6335	93.7	41.6
10. L1-C0	No confirmatory event required	13,810	337.4	195.5	8378	313.7	142.3	9686	143.2	67.2
11. L7-C0	Lookback 7 years, no confirmatory event required	11,728	286.5	169.7	7224	270.5	124.5	8382	123.9	59.1
12. L1-C1-ed	Exclusion of death as confirmatory event	10,135	247.6	137.2	6032	225.9	101.4	5994	88.6	39.5
13. L7-C1-ed	Lookback 7 years, exclusion of death as confirmatory event	9261	226.3	127.7	5478	205.1	93.2	5360	79.2	35.8
14. L1-C4-su	Confirmation 4 quarters, surgery as required confirmatory event	8162	199.4	114.9	N/A	N/A	N/A	5645	83.5	37.1
15. L7-C4-su	Lookback 7 years, confirmation 4 quarters, surgery as required confirmatory event	8015	195.8	113.1	N/A	N/A	N/A	5341	79.0	35.2

Main analysis: All insured persons with a minimum of 7 years of continuous insurance on 01 January 2013

Specification of the algorithms: *L1 to L7* length of lookback period, *C0, C1 and C4* length of confirmation period, *ed* exclusion of death as confirmatory event, *su* surgery as required confirmatory event

### Statistical analysis

In accordance with the ZfKD, crude cumulative incidences (CCI) and age-standardized cumulative incidences (ACI) were estimated, the latter by using the same 1976 European Standard Population. CCI and ACI were compared to ZfKD data [17]. As results from the ZfKD and the Association of Population-based Cancer Registries in Germany (GEKID) indicate that the incidence of cancer diseases differs between the 16 federal states (Länder) of Germany, ACI were stratified by state to compare regional ACI estimates to regional GEKID data [18].

To determine the effect of a changing denominator on ACI estimates, two sensitivity analyses were conducted. First, the study population comprised all insured persons with at least 1 year (instead of 7 years) of continuous insurance on 01 January 2013. Second, the study population comprised all insured persons with a continuous insurance of at least the length of the respective lookback period. The latter analysis resulted in a different denominator for each choice of the lookback period.

All analyses were conducted using SAS 9.4 (SAS Institute Inc., Cary, NC, USA).

## Results

In the main analysis, the study population comprised 6,763,549 insured persons (4,093,251 women and 2,670,298 men) with at least 7 years of continuous insurance. The mean age in 2013 was 51.1 years (52.9 years for women and 48.4 years for men). 31,240 (0.5%) insured persons with at least 7 years of continuous insurance were not included in the study population because of missing or invalid information on sex (*n* = 18), year of birth (*n* = 472) or place of residence (*n* = 30,750). Further 18,884 (0.3%) insured persons were not included since they resided outside of Germany. Insured persons with missing or invalid information on place of residence or places of residence outside of Germany were with an average age of 47.5 years slightly younger than the study population with 51.9 years.

Using the baseline algorithm, 10,312 incident breast cancer cases, 6200 incident prostate cancer cases and 6513 incident colorectal cancer cases were identified (Table 1). By extending the lookback period to 2 years, the number of incident cancer cases declined by 486 (−4.7%), 284 (−4.6%) and 341 (−5.2%), respectively, since these cases had a respective cancer diagnosis in the second year of lookback period and, therefore, were classified as prevalent or recurrent. With a lookback period of 7 years, 8.8% (breast), 9.3% (prostate) and 10.3% (colorectal) fewer incident cancer cases were identified compared to the baseline algorithm.

An extension of the confirmation period from 1 quarter to 4 quarters increased the number of incident breast, prostate and colorectal cancer cases by 887 (+8.6%), 684 (+11.0%) and 700 (+10.7%), respectively. The exclusion of death as a confirmatory event, by contrast, reduced the respective numbers by 177 (−1.7%), 168 (−2.7%) and 519 (−8.0%). When surgery was added as a required confirmatory event during a confirmation period of 4 quarters, the number of incident breast and colorectal cancer cases decreased by 3037 (−27.1%) and 1568 (−21.7%), respectively. When no confirmatory event was required, the number of incident breast, prostate and colorectal cancer cases increased by 3498 (+33.9%), 2178 (+35.1%) and 3173 (+48.7%).

**Fig. 1:**
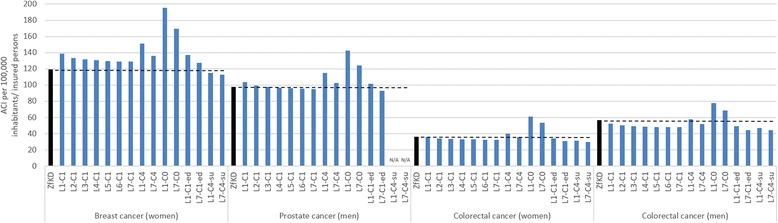
Age-standardized cumulative incidences (ACI) per 100,000 inhabitants presented by the German Centre for Cancer Registry Data (ZfKD) compared to ACI per 100,000 insured persons (claims data, algorithm 1 to 15) for breast, prostate and colorectal cancer in 2013

For breast cancer, the baseline ACI per 100,000 insured persons of 138.7 was 16.3% higher compared to the ZfKD (119.3) (Fig. 1). Although the extension of the lookback period from 1 to 7 years reduced the ACI to 129.0, it was still 8.1% higher compared to the ZfKD. The ACI was also higher when using the longer confirmation period of 4 quarters and still higher when death was excluded as a confirmatory event. When surgery was required as a confirmatory event, the ACI was 3.7% (lookback period 1 year) and 5.2% lower (lookback period 7 years). The ACI were 63.9% and 42.2% higher when no confirmatory event was required.

For prostate cancer, a similar pattern was observed. The estimated baseline ACI of 103.6 was 5.7% higher compared to the ZfKD (98.0). An extension of the lookback period to 7 years reduced the ACI to 95.1, which is 3.0% lower than reported by the ZfKD. When the confirmation period was extended to 4 quarters, ACI were higher compared to ZfKD data. The exclusion of death as a confirmatory event led to ACI which were higher with a lookback period of 1 year but lower with a lookback period of 7 years. When no confirmatory event was required, the ACI were 45.2% and 27.0% higher.

Regarding colorectal cancer, the baseline ACI of 36.0 in women and 52.7 in men were 0.8% and 6.9% lower compared to the ZfKD (women 36.3, men 56.6). Using a lookback period of 7 years reduced the ACI to 32.7 in women and 48.0 in men. By increasing the confirmation period to 4 quarters, in women and men, the ACI were higher with a lookback period of 1 year but lower with a lookback period of 7 years compared to ZfKD data. When surgery was required as a confirmatory event, the ACI in women were 13.2% (lookback period 1 year) and 17.6% (lookback period 7 years) lower compared to the ZfKD. In men, the ACI were 17.1% and 21.6% lower. When no confirmatory event was required, the ACI were 68.3% (women) and 38.0% (men) higher with a lookback period of 1 year and 47.9% and 21.6% higher with a lookback period of 7 years.

Our regional ACI estimates for breast, prostate and colorectal cancer indicated similar regional variations when compared to GEKID data (Table 2).

**Table 2:** Age standardized cumulative incidences (ACI) per 100,000 inhabitants in cancer registries compared to ACI per 100,000 insured persons in claims data for breast, prostate and colorectal cancer in 2013 stratified by the 16 federal states (Länder) of Germany

Federal State (Land)	ACI per 100,000 insured persons/ inhabitants											
	Breast cancer (women)			Prostate cancer (men)			Colorectal cancer (women)			Colorectal cancer (men)		
	Claims data (L7_C1)	ZfKD/GEKID	Diff. [%]	Claims data (L7_C1)	ZfKD/GEKID	Diff. [%]	Claims data (L7_C4)	ZfKD/GEKID	Diff. [%]	Claims data (L7_C4)	ZfKD/GEKID	Diff. [%]
Germany (total)	129.0	119.3	8.1	95.1	98.0	−3.0	35.8	36.3	−1.4	51.7	56.6	−8.7
Baden-Württemberg	133.9	-	-	101.2	-	-	35.0	-	-	48.1	-	-
Bavaria	125.5	108.6	15.6	100.9	93.2	8.3	33.6	33.8	−0.7	51.9	56.2	−7.6
Berlin	125.8	111.8	12.5	76.6	48.5	58.0	34.6	28.4	22.0	55.5	42.7	30.0
Brandenburg	117.6	99.6	18.1	97.5	91.7	6.3	37.6	33.6	12.0	57.2	56.2	1.7
Bremen	108.2	124.8	−13.3	68.6	84.4	−18.7	46.1	36.0	28.2	36.6	54.1	−32.3
Hamburg	137.4	136.2	0.9	94.3	87.7	7.6	45.8	41.9	9.4	53.5	58.0	−7.8
Hesse	123.1	136.3	−9.7	89.9	95.5	−5.8	34.7	36.5	−5.0	50.1	57.7	−13.2
Lower Saxony	134.8	129.3	4.3	95.2	107.3	−11.3	34.4	38.6	−10.9	46.2	57.1	−19.1
Mecklenburg-Western- Pomerania	119.9	106.7	12.4	93.8	92.5	1.5	35.4	34.2	3.5	39.4	55.9	−29.6
North-Rhine-Westphalia	140.6	130.1	8.1	91.2	99.2	−8.1	38.6	39.8	−3.0	53.4	57.2	−6.7
Rhineland-Palatinate	129.7	116.3	11.5	95.8	87.8	9.1	39.3	33.8	16.3	51.6	51.5	0.2
Saarland	129.0	116.6	10.7	86.8	78.4	10.7	45.1	37.7	19.7	55.3	62.0	−10.8
Saxony	108.7	103.5	5.0	100.0	89.7	11.5	30.6	31.7	−3.4	59.2	55.9	6.0
Saxony-Anhalt	99.1	95.7	3.6	102.6	68.1	50.6	26.4	27.5	−3.9	44.2	52.1	−15.1
Schleswig-Holstein	151.7	130.8	16.0	99.1	103.1	−3.9	40.8	38.8	5.2	50.9	51.9	−2.0
Thuringia	101.9	96.3	5.8	103.2	92.2	11.9	30.3	34.0	−10.9	62.9	57.9	8.7

Main analysis: All insured persons with a minimum of 7 years of continuous insurance on 01 January 2013

*ZfKD* German Centre for Cancer Registry Data, ACI in Germany (total) [17], *GEKID* Association of Population-based Cancer Registries in Germany, ACI in the 16 federal states of Germany [18]

The estimations of the CCI and ACI were robust to the changes in the sensitivity analyses (Additional file 1).

## Discussion

We systematically examined the impact of the length of the lookback period, the length of the confirmation period and the definition of confirmatory events on the number of incident cancer cases identified and ACI estimated in claims data using cancer registry data as a benchmark. We applied 15 algorithms and found that the number of incident cancer cases identified and ACI estimated varied considerably depending on the algorithm used.

The number of identified incident breast, prostate and colorectal cancer cases declined substantially with a lookback period of 2 or more years instead of only 1 year, indicating a large number of false positives when using the shortest lookback period. We believe that the principal reasons for the declining number of incident cancer cases are (1) prevalent cancer cases that decided to forego treatment options financed by the SHI, (2) recurrent cancer cases with a period between onset and recurrence of the disease longer than the respective lookback period and (3) prevalent cancer cases that are treated by active surveillance or watchful waiting (particularly in the case of prostate cancer). A large proportion of these false positives could already be eliminated by increasing the lookback period from 1 to 2 years. Therefore, we discourage from using lookback periods of 1 year which are often the standard and suggest using lookback periods of 2 or more years. Recommendations to use longer lookback periods have also been made for other diseases [15, 19, 20] and drug prescriptions [21].

Using a population with no respective cancer diagnosis in a lookback period of 7 years, we showed that increasing the confirmation period from 1 to 4 quarters resulted in more incident cases. The gain in confirmed cases was even higher in a population with no respective cancer diagnosis in a lookback period of 1 year. These findings indicate that, first, a confirmation period of 1 quarter may be too short to confirm all incident cases and second, the proportion of false positives may increase when extending the confirmation period. It is, furthermore, important to consider that the coding frequency of diagnoses may differ by patients’ behavior, tumor type and probably also by tumor stage. More aggressive tumors may be treated sooner and more frequently and thus related diagnostic codes will be seen earlier whereas less aggressive tumors may be treated differently, e.g. treatment by active surveillance or watchful waiting in the case of prostate cancer [22]. We therefore suggest applying longer confirmation periods for less aggressive cancers, but more strict criteria for aggressive cancers.

Concerning the definition of confirmatory events, the exclusion of death as a confirmatory event slightly reduced the number of incident breast and prostate cancer cases which have a lower lethality. For incident colorectal cancer cases, which have a higher lethality, the observed reduction was more than twice as high. Therefore, we suggest considering death as a potential confirmatory event when identifying incident cases for cancer sites which have a higher lethality. The inclusion of breast and colorectal surgeries as a required confirmatory event in a confirmation period of 4 quarters reduced the number of incident cases dramatically by a quarter (breast) and a fifth (colorectal). This is in line with previous results [6], which showed that the inclusion of surgeries reduced the number of false positive incident breast cancer cases but lowered sensitivity substantially. Reportedly, depending on the characteristics of the patient and the tumor, a non-negligible proportion of primary cancer cases does not receive surgical treatment (breast), is treated by active surveillance or watchful waiting (prostate) or is treated non-operatively by palliative or curative care (colorectal) [22–24]. Therefore, the inclusion of surgery as a required confirmatory event may result in a significant number of incident cancer cases not being identified. On the other hand, when confirmatory events were not required at all, the number of incident cases increased dramatically which may suggest a large number of false positives. We thus recommend confirming incident cancer diagnoses using confirmation periods of at least 1 quarter. Both the exact length of the confirmation period and the definition of confirmatory events should be defined according to the characteristics of the specific cancer site, taking account of the available data and the underlying research question. For example, outcomes research studies may try to increase specificity, whereas registry validation studies may focus on achieving high sensitivity [7].

When compared to the ZfKD, our claims-based baseline ACI were higher for breast and prostate cancer with higher 5-year survival rates but lower for colorectal cancer with a lower 5-year survival rate [16, 17]. By increasing the lookback period, we obtained better comparability for breast and prostate cancer. For colorectal cancer, however, the discrepancy increased. When breast and colorectal surgeries were included in the algorithm as a required confirmatory event, our ACI estimates were considerably lower. When confirmatory events were not required at all, the ACI estimates increased dramatically suggesting a relatively high number of false positives. Stratified by the 16 federal states of Germany, our regional ACI estimates showed trends similar to GEKID data [18].

Interestingly, Charlton et al. [25] also showed lower incidence estimates for colorectal cancer on the General Practice Research Database (GPRD) in comparison to those in national cancer registries. Similar results were observed by Haynes et al. in the Health Improvement Network (THIN) database [26] while others reported incidence estimates for various cancers which were more in line with cancer registries [27–31]. However, comparability between study results is limited, especially due to differences in study designs, coding systems and claims data used.

Overall, comparing our ACI estimates to cancer registry data strengthened our recommendations to use longer lookback periods as well as to adapt both the length of the confirmation period and the definition of confirmatory events to the characteristics of the specific cancer site, the characteristics of the available data and the research question examined.

To achieve better comparability between ACI estimates obtained by our 15 algorithms, we eliminated influences of a changing denominator by using the same cohort throughout the whole main analysis. To determine the effect of a changing denominator, we conducted two sensitivity analyses. First, only 1 year of continuous insurance before cohort entry was required. However, for insured persons with longer continuous insurance, all available information in the respective lookback period was considered, similar as recommended by Gilbertson et al. [32]. The resulting ACI estimates were slightly lower compared to the main analysis when longer lookback periods were used. This finding probably resulted from the larger denominator. On the other hand, this approach might have resulted in a higher proportion of false-positives, as cases with a prior diagnosis of the respective cancer site before the start of availability of information would have been falsely counted as incident. In epidemiology, cohort inclusion criteria usually depend on the length of the defined lookback period. We therefore performed a second sensitivity analysis in which the study population comprised all insured persons with a continuous insurance of at least the length of the lookback period used in the respective algorithm and found almost identical ACI estimates compared to the main analysis.

### Strengths and limitations

This is the first study that systematically examined the impact of the length of the lookback period, the length of the confirmation period and the definition of confirmatory events on the number of incident cancer cases identified and cumulative incidences estimated in administrative claims data. The large sample size allowed us to estimate CCI and ACI with good precision and the long observation period enabled us to apply lookback and confirmation periods of various lengths. As the data source comprised in- and outpatient claims data, incident cancer cases could be identified in both settings. Furthermore, diagnoses, deaths and surgeries could be considered when identifying incident cancer cases.

Due to data protection, however, it was not possible to link administrative claims data to cancer registry data. Therefore, we were not able to estimate sensitivities and PPV. Moreover, we were unable to examine the extent of misclassification and our incidence estimates may be susceptible to compensating errors [33, 34]. In this case, an equal number of false positives and false negatives could have resulted in ACI comparable to those observed in cancer registries. However, the development of valid algorithms for the identification of incident cancer cases was beyond the scope of our study. Moreover, a poor sensitivity for identifying death certificate only cases in administrative claims data has been reported [19] which might have lowered our ACI estimates. Finally, the generalizability of our results is limited, particularly because of structural differences between SHI funds [35]. However, the ACI presented by the ZfKD are estimates based on the numbers or expected values of the regional cancer registries and thus have some limitations, too. Despite these methodological issues regarding completeness of data, the ZfKD estimates still provide a valid benchmark.

## Conclusions

The choice of the length of the lookback period, the length of the confirmation period and the definition of confirmatory events have a considerable impact on the number of incident cancer cases identified and ACI estimated. It is not possible to give general recommendations, as the optimal algorithm depends on the characteristics of the specific cancer site, the characteristics of the available data and the underlying research question. However, we discourage from using lookback periods of 1 year and recommend using lookback periods of 2 or more years. Moreover, we recommend confirming incident cancer diagnoses using confirmation periods of at least 1 quarter. In the light of our findings, we advise to carefully consider which algorithm to use and to clearly describe how incident cases were identified. Further validation studies as well as studies using administrative data to estimate cancer incidences should consider several choices of the lookback and confirmation periods and the definition of confirmatory events to show how these parameters impact the validity and robustness of their results.

## Additional file

Additional file 1: Results of the sensitivity analyses. (PDF 429 kb)

## Abbreviations

ACI: Age-standardized cumulative incidence(s)
CCI: Crude cumulative incidence(s)
EBM: German uniform assessment standard
GEKID: Association of Population-based Cancer Registries in Germany
GPRD: General Practice Research Database
ICD-10-GM: International classification of diseases, 10th revision, German modification
OPS: German Procedure Classification
PPV: Positive predictive value(s)
SHI: Statutory Health Insurance
THIN: The Health Improvement Network
ZfKD: German Centre for Cancer Registry Data
