# Supplementary material for: The impact of the lookback period and definition of confirmatory events on the identification of incident cancer cases in administrative data
Source: BMC Med Res Methodol. 2017 Aug 14;17:122. doi: 10.1186/s12874-017-0407-4 (PMC5556662; doi:10.1186/s12874-017-0407-4)
Supplement: Additional file 1: — Results of the sensitivity analyses. (PDF 429 kb) [file 12874_2017_407_MOESM1_ESM.pdf]

Additional file 1: Results of the sensitivity analyses

The impact of the lookback period and definition of confirmatory events on the identification of incident cancer cases in administrative data

BMC Medical Research Methodology

Jonas Czwikla[1,2], Kathrin Jobski[3], Tania Schink[4]

[1] Department of Health, Long-term Care and Pensions, SOCIUM Research Center on Inequality and Social Policy, University of Bremen, P.O. Box 33 04 40, 28334 Bremen, Germany

[2] High-Profile Area Health Sciences, University of Bremen, P.O. Box 33 04 40, 28334 Bremen, Germany

[3] Department of Health Services Research, Carl von Ossietzky University of Oldenburg, P.O. Box 2503, 26111 Oldenburg, Germany

[4] Leibniz Institute for Prevention Research and Epidemiology - BIPS, Drug Safety Unit, Achterstrasse 30, 28359 Bremen, Germany

Corresponding author

Jonas Czwikla, M.A.

Co-authors

Dr. rer. nat. Kathrin Jobski

Dr. rer. medic. Tania Schink

Description

We conducted two sensitivity analyses. In the first analysis (results are shown in Table A.1 and Figure A.1), the study population comprised all insured persons with at least 1 year (instead of 7 years) of continuous insurance on 01 January 2013. In the second analysis (results are shown in Table A.2 and Figure A.2), the study population comprised all insured persons with a continuous insurance of at least the length of the respective lookback period on 01 January 2013.

Table A.1: Numerator (n), crude cumulative incidences (CCI) and age-standardized cumulative incidences (ACI) per 100,000 insured persons for breast, prostate and colorectal cancer in 2013 (sensitivity analysis 1).

Algorithm	Description	Breast cancer (women) Denominator: n = 4,973,849			Prostate cancer (men) Denominator: n = 3,534,427			Colorectal cancer (overall) Denominator: n = 8,508,276		
		Num. (n)	CCI per 100,000	ACI per 100,000	Num. (n)	CCI per 100,000	ACI per 100,000	Num. (n)	CCI per 100,000	ACI per 100,000
1. L1-C1	Baseline algorithm, lookback 1 year, confirmation 1 quarter	11,164	224.5	137.0	6,667	188.6	103.8	6,987	82.1	42.4
2. L2-C1	Lookback 2 years	10,580	212.7	130.8	6,335	179.2	99.1	6,585	77.4	40.1
3. L3-C1	Lookback 3 years	10,317	207.4	127.7	6,167	174.5	96.6	6,394	75.2	39.1
4. L4-C1	Lookback 4 years	10,164	204.3	125.9	6,083	172.1	95.2	6,299	74.0	38.5
5. L5-C1	Lookback 5 years	10,054	202.1	124.6	6,021	170.4	94.3	6,223	73.1	38.0
6. L6-C1	Lookback 6 years	9,955	200.1	123.2	5,929	167.7	92.8	6,147	72.2	37.4
7. L7-C1	Lookback 7 years	9,845	197.9	121.6	5,875	166.2	91.9	6,097	71.7	37.1
8. L1-C4	Confirmation 4 quarters	12,145	244.2	149.6	7,388	209.0	114.9	7,739	91.0	47.1
9. L7-C4	Lookback 7 years, confirmation 4 quarters	10,339	207.9	128.3	6,346	179.5	99.3	6,614	77.7	40.3
10. L1-C0	No confirmatory event required	15,083	303.2	193.0	9,012	255.0	142.1	10,439	122.7	67.2
11. L7-C0	Lookback 7 years, no confirmatory event required	12,344	248.2	159.0	7,558	213.8	120.0	8,775	103.1	56.7
12. L1-C1-ed	Exclusion of death as confirmatory event	10,983	220.8	135.6	6,496	183.8	101.7	6,453	75.8	39.8
13. L7-C1-ed	Lookback 7 years, exclusion of death as confirmatory event	9,695	194.9	120.4	5,728	162.1	90.1	5,600	65.8	34.7
14. L1-C4-su	Confirmation 4 quarters, surgery as required confirmatory event	8,878	178.5	113.3	N/A	N/A	N/A	6,072	71.4	37.4
15. L7-C4-su	Lookback 7 years, confirmation 4 quarters, surgery as required confirmatory event	8,394	168.8	106.5	N/A	N/A	N/A	5,572	65.5	34.0

Sensitivity analysis 1: All insured persons with a minimum of 1 year of continuous insurance on 01 January 2013.

Specification of the algorithms: L1 to L7 = length of lookback period; C0, C1 and C4 = length of confirmation period; ed = exclusion of death as confirmatory event; su = surgery as required confirmatory event.

Table A.2: Numerator (n), denominator (n), crude cumulative incidences (CCI) and age-standardized cumulative incidences (ACI) per 100,000 insured persons for breast, prostate and colorectal cancer in 2013 (sensitivity analysis 2).

Algorithm	Description	Breast cancer (women)				Prostate cancer (men)				Colorectal cancer (overall)			
		Num. (n)	Denom. (n)	CCI per 100,000	ACI per 100,000	Num. (n)	Denom. (n)	CCI per 100,000	ACI per 100,000	Num. (n)	Denom. (n)	CCI per 100,000	ACI per 100,000
1. L1-C1	Baseline algorithm, lookback 1 year, confirmation 1 quarter	11,164	4,973,849	224.5	137.0	6,667	3,534,427	188.6	103.8	6,987	8,508,276	82.1	42.4
2. L2-C1	Lookback 2 years	10,470	4,786,780	218.7	132.1	6,283	3,356,505	187.2	99.9	6,535	8,143,285	80.3	40.5
3. L3-C1	Lookback 3 years	10,127	4,614,898	219.4	130.6	6,071	3,191,477	190.2	97.8	6,290	7,806,375	80.6	39.6
4. L4-C1	Lookback 4 years	9,902	4,474,085	221.3	129.7	5,946	3,046,058	195.2	96.7	6,165	7,520,143	82.0	39.2
5. L5-C1	Lookback 5 years	9,729	4,351,412	223.6	129.2	5,857	2,925,796	200.2	96.2	6,062	7,277,208	83.3	39.0
6. L6-C1	Lookback 6 years	9,571	4,222,455	226.7	129.1	5,713	2,796,503	204.3	95.3	5,931	7,018,958	84.5	38.4
7. L7-C1	Lookback 7 years	9,409	4,093,251	229.9	129.0	5,623	2,670,298	210.6	95.1	5,842	6,763,549	86.4	38.3
8. L1-C4	Confirmation 4 quarters	12,145	4,973,849	244.2	149.6	7,388	3,534,427	209.0	114.9	7,739	8,508,276	91.0	47.1
9. L7-C4	Lookback 7 years, confirmation 4 quarters	9,866	4,093,251	241.0	136.0	6,077	2,670,298	227.6	102.9	6,335	6,763,549	93.7	41.6
10. L1-C0	No confirmatory event required	15,083	4,973,849	303.2	193.0	9,012	3,534,427	255.0	142.1	10,439	8,508,276	122.7	67.2
11. L7-C0	Lookback 7 years, no confirmatory event required	11,728	4,093,251	286.5	169.7	7,224	2,670,298	270.5	124.5	8,382	6,763,549	123.9	59.1
12. L1-C1-ed	Exclusion of death as confirmatory event	10,983	4,973,849	220.8	135.6	6,496	3,534,427	183.8	101.7	6,453	8,508,276	75.8	39.8
13. L7-C1-ed	Lookback 7 years, exclusion of death as confirmatory event	9,261	4,093,251	226.3	127.7	5,478	2,670,298	205.1	93.2	5,360	6,763,549	79.2	35.8
14. L1-C4-su	Confirmation 4 quarters, surgery as required confirmatory event	8,878	4,973,849	178.5	113.3	N/A	N/A	N/A	N/A	6,072	8,508,276	71.4	37.4
15. L7-C4-su	Lookback 7 years, confirmation 4 quarters, surgery as required confirmatory event	8,015	4,093,251	195.8	113.1	N/A	N/A	N/A	N/A	5,341	6,763,549	79.0	35.2

Sensitivity analysis 2: All insured persons with a continuous insurance of at least the length of the respective lookback period on 01 January 2013.

Specification of the algorithms: L1 to L7 = length of lookback period; C0, C1and C4 = length of confirmation period; ed = exclusion of death as confirmatory event; su = surgery as required confirmatory event.

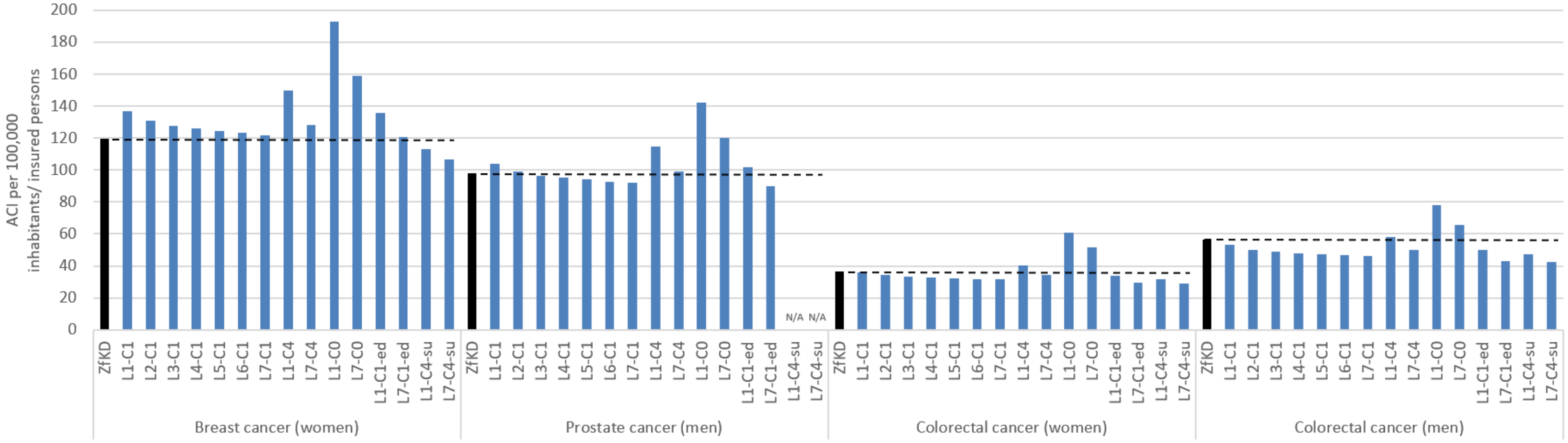

Figure A.1: Age-standardized cumulative incidence (ACI) per 100,000 inhabitants presented by the German Centre for Cancer Registry Data (ZfKD) compared to ACI per 100,000 insured persons (claims data, algorithm 1 to 12) for breast, prostate and colorectal cancer in 2013 (sensitivity analysis 1).

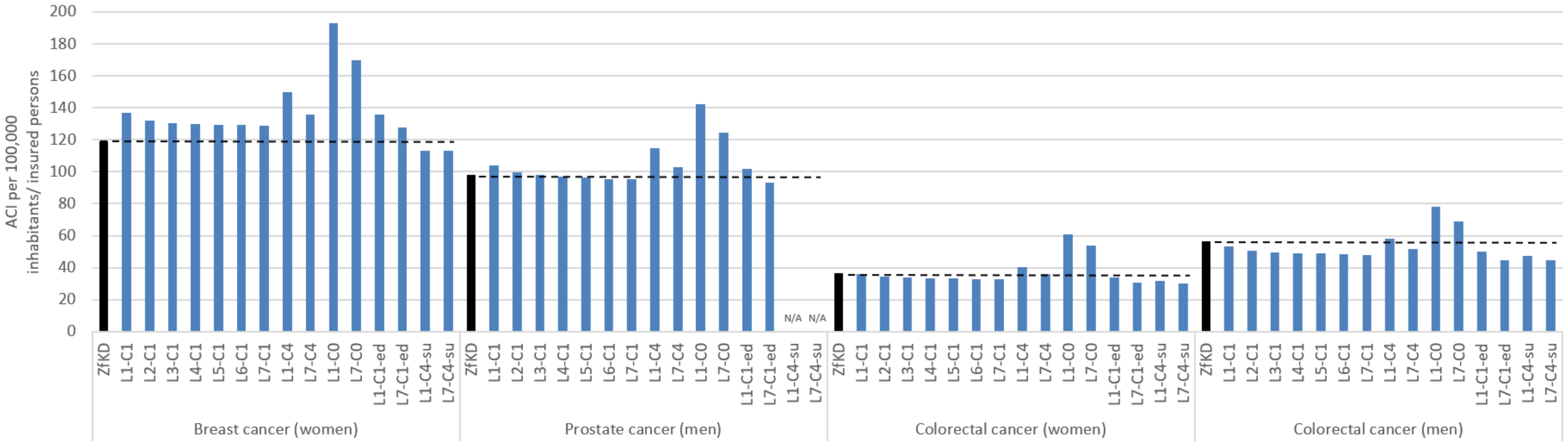

Figure A.2: Age-standardized cumulative incidence (ACI) per 100,000 inhabitants presented by the German Centre for Cancer Registry Data (ZfKD) compared to ACI per 100,000 insured persons (claims data, algorithm 1 to 12) for breast, prostate and colorectal cancer in 2013 (sensitivity analysis 2).
